# An oral care programme for adults- Evaluation after 15 years

**DOI:** 10.1371/journal.pone.0223960

**Published:** 2019-12-05

**Authors:** Carolina Ganss, Marie Heins, Nadine Schlueter

**Affiliations:** 1 Department of Conservative and Preventive Dentistry, Dental Clinic of the Justus-Liebig-University Giessen, Giessen, Germany; 2 Division for Cariology, Department of Operative Dentistry and Periodontology, Center for Dental Medicine, Medical Center—University of Freiburg, Faculty of Medicine, University of Freiburg, Freiburg, Germany; University of Bern, SWITZERLAND

## Abstract

The present retrospective analysis sought to investigate the impact of the oral care programme (OCP) for adults provided at the Department of Conservative and Preventive Dentistry, Justus-Liebig-University of Giessen, Germany, on oral health parameters. The OCP was modular and included oral hygiene instruction/professional toothcleaning, nutrition counselling, fluoridation and re-motivation. From 1999–2014, data from 1665 patients (55.1% female, 44.9% male; median age 33 years, range 15;80) were available. Type/date of modules, % of proximal sites with plaque (PP) and with bleeding after probing (PB) and D_3/4_MFT/D_3/4_MFS were recorded. PP and PB values are given as median (min;max). Overall, 60.2% of the patients attended the OCP once, 19.1% twice, and 20.7% ≥ three times. Initially, PP/PB were 0.68(0;1)/0.08(0;1) resp. decreasing at visit two (0.62(0;1)/0.07(0;1) resp.; p≤0.001 each) with no further improvement over next visits. Patients with poor oral hygiene improved, but those with good oral hygiene worsened (p≤0.001 each). Shorter intervals between visits were more effective than longer intervals. Attendance patterns changed significantly over the years: earlier, patients attended more visits with different modules; later, patients preferred the oral hygiene module and the intervals between visits lengthened. Prevalence and incidence of caries was associated with higher PP levels. Attendance patterns changed over time and had a significant impact on the outcome of the OCP. The improvement of oral hygiene was limited and occurred within the first two visits; repeated sessions maintained this improvement. The results indicate the need for new strategies improving patients’ skills for efficient hygiene techniques.

## Introduction

It is known for long that caries, gingivitis and periodontitis are plaque-associated oral diseases. Early studies using rat models clearly showed that caries doesn’t appear in the absence of microorganisms, clarified the role of sucrose in the caries process and demonstrated the benefits of local fluoride exposures [1–3]. Later, the human studies of Loe and van der Fehr highlighted the role of oral hygiene for the development of gingivitis and caries and the importance of sugar for the development of caries [4–6]. Since these times, basic knowledge is available for all aspects of the aetiology of plaque-associated diseases constituting current prevention concepts. The best documented preventive measure is the administration of fluorides [7–9] whereas the role of other measures like nutrition counselling, oral hygiene instruction or oral health education is much less clear [10–12].

Up to now many different intervention programmes have been initiated, the overwhelming majority addressing children and adolescents. Regarding adulthood, there are some studies on older adults published [13], but almost nothing is known about effects of prevention programmes in younger and middle aged adults. However, effects from interventions in young or old aged groups cannot be simply extrapolated to other age groups because psychological and cognitive competences as well as motor abilities change significantly over the life course, as do social contexts. All these conditions affect oral health risks.

Axelsson and co-workers reported about a prevention program (the so-called Karlstad model) starting in 1971 and continuing for 30 years [14–17]. It included case presentation and education, self-diagnosis and self-care with recall visits every two months in the first two years. In the years three to six, recall visits were held once every three months, followed by a risk based recall frequency for the rest of the years. After six years, participants of the test group had markedly improved their plaque and gingivitis values and except for few individuals, no caries occurred. This was in clear contrast to results for subjects in the control group who did not improve with respect to oral hygiene values and developed many new caries lesions in the same time period [15]. These results led to the exclusion of the control group. After 30 years, the number of new decayed surfaces was between 1.2 and 2.1 depending on the risk group, and subjects exhibited more than 90% healthy periodontal sites.

The Karlstad model provided intensive care with frequent recall visits, which may not be suitable for broader use. Therefore, two other types of programmes with three initial visits and yearly recalls were compared to the Karlstad model in a randomised controlled study by Hugoson et al. [18]. All kinds of programmes improved plaque and gingivitis levels compared to no intervention, but there were no significant differences between them.

In view of the lack of knowledge about effects of prevention programmes in adults, the present study sought to retrospectively investigate the outcome of our Oral Care Programme (OCP). It was structured in four different modules and performed in our clinic for more than 15 years. The modules addressed oral hygiene education, nutrition counselling, fluoridation and re-motivation and were performed by dental students supervised by dentists.

The aim of the study was to evaluate the patterns of attendance at the OCP, the effects of the OCP on oral hygiene and on caries, and how the patterns of attendance influenced these effects.

## Patients, materials and methods

The study is a retrospective analysis of patient paper recordings from the years 1999–2014 and was approved by the Ethics Committee of the Justus-Liebig-University, Giessen, Germany (Doc. No 104/15).

### Structure of the oral care programme

The OCP took place at the Department for Conservative and Preventive Dentistry of the Justus-Liebig-University Giessen. It was performed by fourth-year dental students supervised by dentists and was part of the routine oral care procedures. The OCP addressed adults, but patients younger than legal age were not explicitly excluded. Patients were included when eligible for being treated by students (no serious infectious illness, e.g. hepatitis or HIV, no serious physical or mental disability). They either were referred to the OCP during restorative dental treatment in our department or contacted us from outside.

The OCP consisted of four modules addressing oral hygiene, nutrition counselling, fluoridation and re-motivation (Table 1). The students had to perform according to a manual exactly describing content and procedures of each module and were supervised by dentists. All procedures performed during a session were documented in the patient record, plaque and gingival bleeding were recorded on standardised paper forms added to the patient record. Patients entered the OCP with the first module. Some patients followed the full course of the OCP attending all modules, but some patients preferred to join only single parts of the OCP depending on individual needs and preferences. At the end of each appointment, the supervising dentist discussed the individual needs for oral care with the patient and made a recommendation as to next steps.

**Table 1:** Content of the modules of the Oral Care Programme.

Module 1: Oral Hygiene	Disclose plaque (erythrosine), record PP and PB, show the patient amounts and location of plaque, explain the role of plaque for oral health, demonstrate toothbrushing and interdental hygiene, perform professional toothcleaning, encourage the use of a fluoridated toothpaste
Module 2: Nutrition Counselling	Disclose plaque (erythrosine), record PP and PB, show the patient persisting amounts and location of plaque and re-motivate, explain the role of sugar for plaque metabolism and growth for caries, take dietary history/dietary diary and recommend healthy diet, perform professional toothcleaning
Module 3: Fluoridation	Disclose plaque (erythrosine), record PP and PB, show the patient persisting amounts and location of plaque and re-motivate, explain the role of fluoride for caries prevention, take fluoride history and recommend fluoridation measures, perform professional toothcleaning
Module 4: Re-motivation	Disclose plaque (erythrosine), record PP and PB, show the patient persisting amounts and location of plaque and re-motivate; individualised module referring to patient’s needs

For scoring the oral hygiene status, the presence/absence of proximal plaque (PP) on the oral and vestibular sites of all teeth was recorded, except third molars. The PP value is the percentage value of proximal sites with plaque of all sites investigated.

Further, the absence/presence of proximal bleeding (PB) after probing the oral and vestibular sites of all teeth except third molars was recorded. The PB value is the percentage value of proximal sites with bleeding of all sites investigated.

Recommendations were given in an individualised manner taking care of the patient´s needs. Toothbrushing was demonstrated on models and regarded the type of toothbrush (manual or power) the patient normally used. For manual brushing, the modified Bass-technique was recommended; patients with limited motor skills should use a powered toothbrush. For all patients special emphasis was put on brushing in a systematic way in order to reach all areas of the dentition. For interdental hygiene, floss was recommended for patients with narrow interdental spaces. Patients with wider interdental spaces and/or bridgework received demonstration of interdental brushes after choosing the adequate size. Patient´s pre- or post-instruction brushing or interdental cleaning performance was not observed. Nutrition counselling included recommending the reduction of snacking and overall sugar consumption as well as replacing sugar by sweeteners/sugar alcohols. Fluoride measures were recommended according to the German Fluoridation Guideline [19].

The general advice for all patients was brushing twice daily with a fluoridated toothpaste (1000–1450 ppm). In cases of increased caries risk a fluoride gel once per week, alternatively a fluoride rinse on a daily basis was recommended.

### Data extraction and statistics

Data were extracted from the complete archived records from 1999–2014 including 1665 patients. As all available records were used, there were no inclusion/exclusion criteria. Age and gender of the patient was recorded, as names and birthdays were excluded, it was not possible to re-identify the patient from the extracted data. Type and date of the modules attended and PP/PB at each visit were recorded. In addition, from 747 patients, D_3/4_MFT and D_3/4_MFS values were available from routine dental treatment in our department.

Data was stratified as to time periods (1999–2004, 2005–2009, 2010–1014), patients were stratified in those with good (PP ≤0.5), fair (PP >0.5 to ≤0.75) and poor (PP >0.75) oral hygiene at the first visit. Instruction intervals were stratified in ≤4 weeks, >4 ≤8 weeks, >8 weeks≤1 year and >1 year.

Data from paper records were transferred to Excel 2010 data sheets (Microsoft Corporation, Redmond, WA, USA). Statistical procedures were performed by using IBM SPSS Statistics, Version 21 (IBM Corporation, Armonk, NY, USA). As there was a significant deviation from the Gaussian distribution (Kolmogorov-Smirnov test) for some of the parameters under study, values are generally given as median (min;max). Comparisons of plaque, gingivitis and caries values are made with nonparametric procedures (Wilcoxon-test for dependent, Mann-Whitney-U-test for independent comparisons). The utilization of the OCP (number of visits, type of modules, interval lengths) was analysed by cross tabulation and chi-square tests. The significance level was set at 0.05.

## Results

### Patients and patterns of attendance

**Fig 1:**
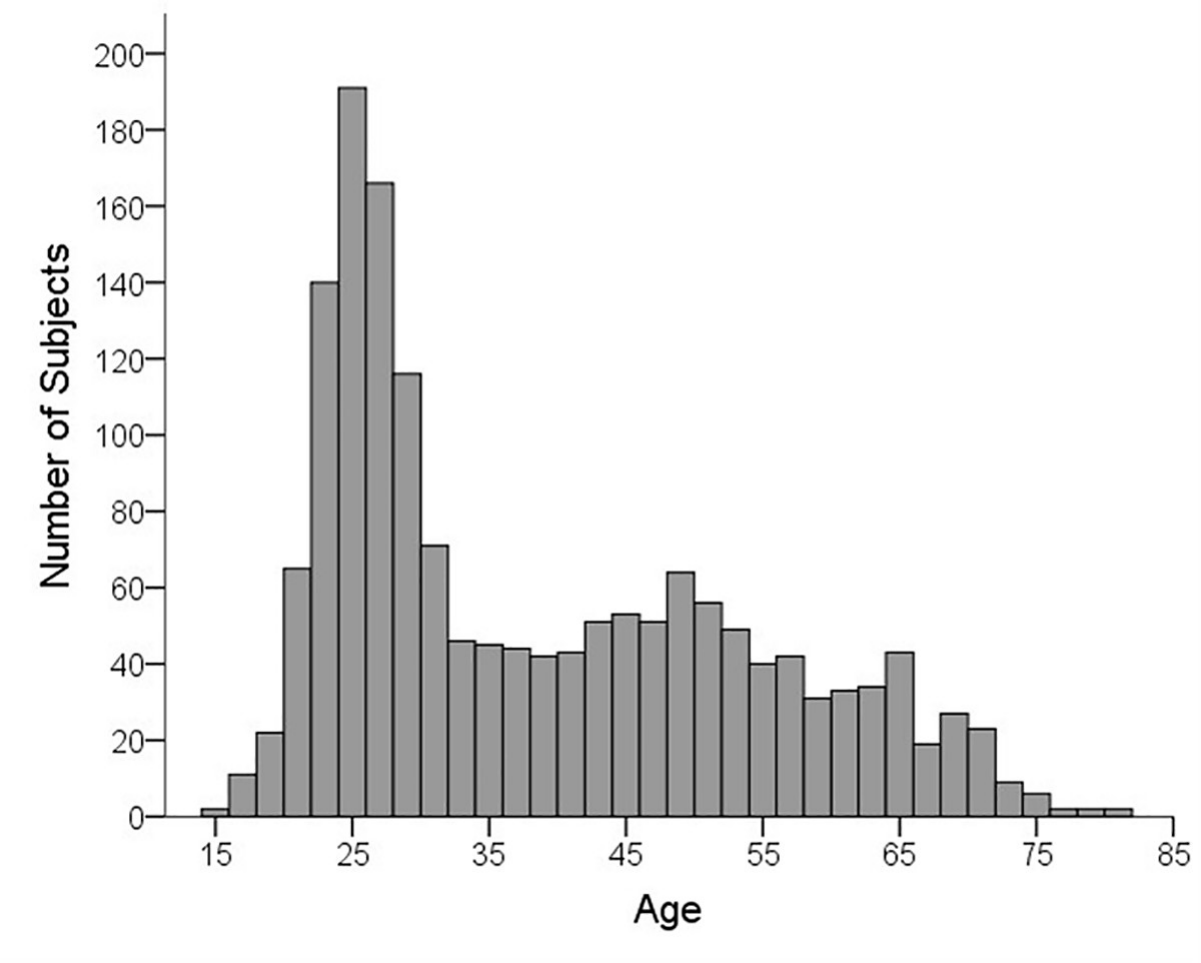
Age distribution of patients attending the OCP.

Among the 1665 patients, 55.1% were female and 44.9% were male. The median age was 33 (15;80), the age distribution is shown in Fig 1.

**Fig 2:**
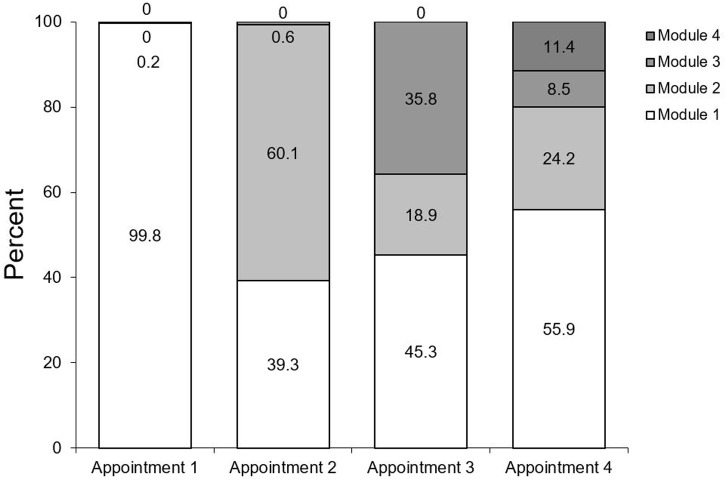
Percentage of module types according to visits.

Overall, 60.2% attended the OCP only once, 19.1% twice, 8% three times and 12.7% four times or more (max. 38). According to the structure of the OCP, the type of the module varied significantly (p≤0.001) depending on the number of visits (Fig 2).

Almost all patients attending the OCP for the first time used oral hygiene module (module 1), at the second visit 60.1% used nutrition counselling module (module 2) and at the third visit 35.8% used fluoride module (module 3).

**Fig 3:**
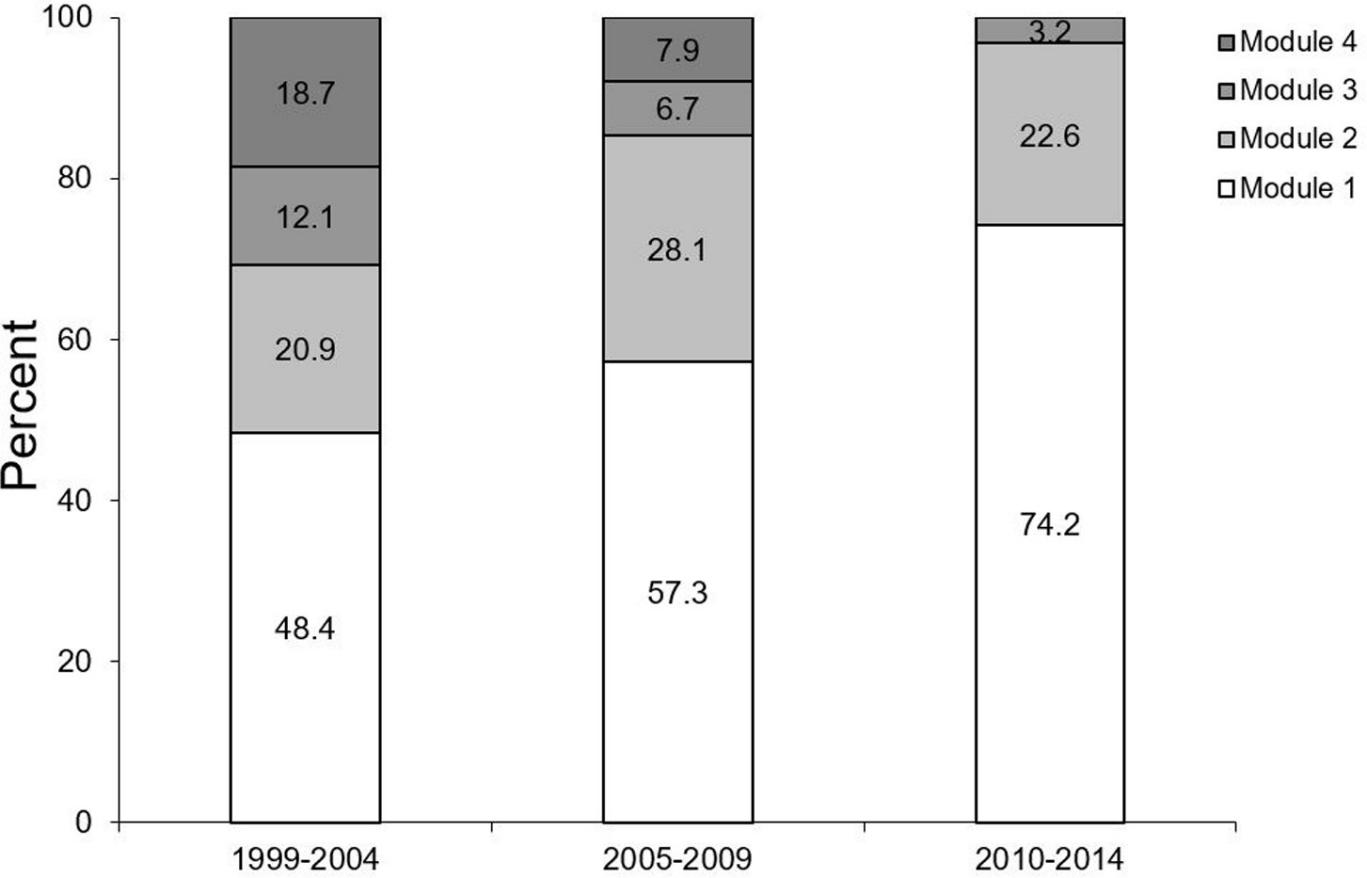
Percentage of module types according to the different time periods (data from patients attending the OCP at least four times).

When looking at the different time periods, a distinct change occurred (Fig 3). Generally, the oral hygiene module was most frequently used, but its percentage of all modules increased distinctly over the years. The opposite was the case for the fluoridation and re-motivation modules, whereas the percentage of using the module addressing nutrition remained relatively stable (p≤0.001).

**Fig 4:**
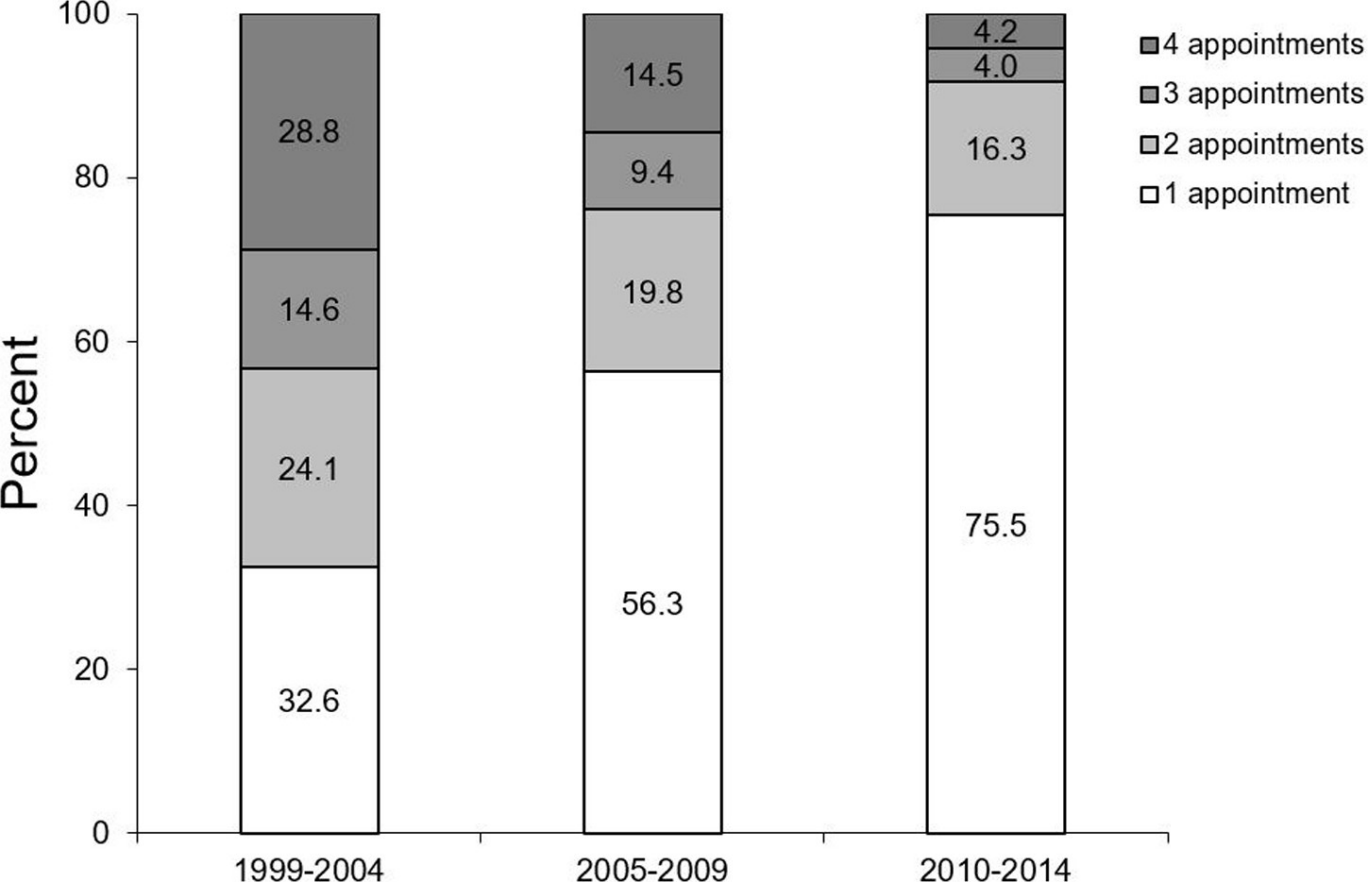
Percentage of numbers of visits according to the different time periods.

Similar results were observed regarding the number of visits per patient (Fig 4). The number of patients attending the OCP only once increased distinctly over time, vice versa, particularly the percentage of patients attending three times or more decreased (p≤0.001).

**Fig 5:**
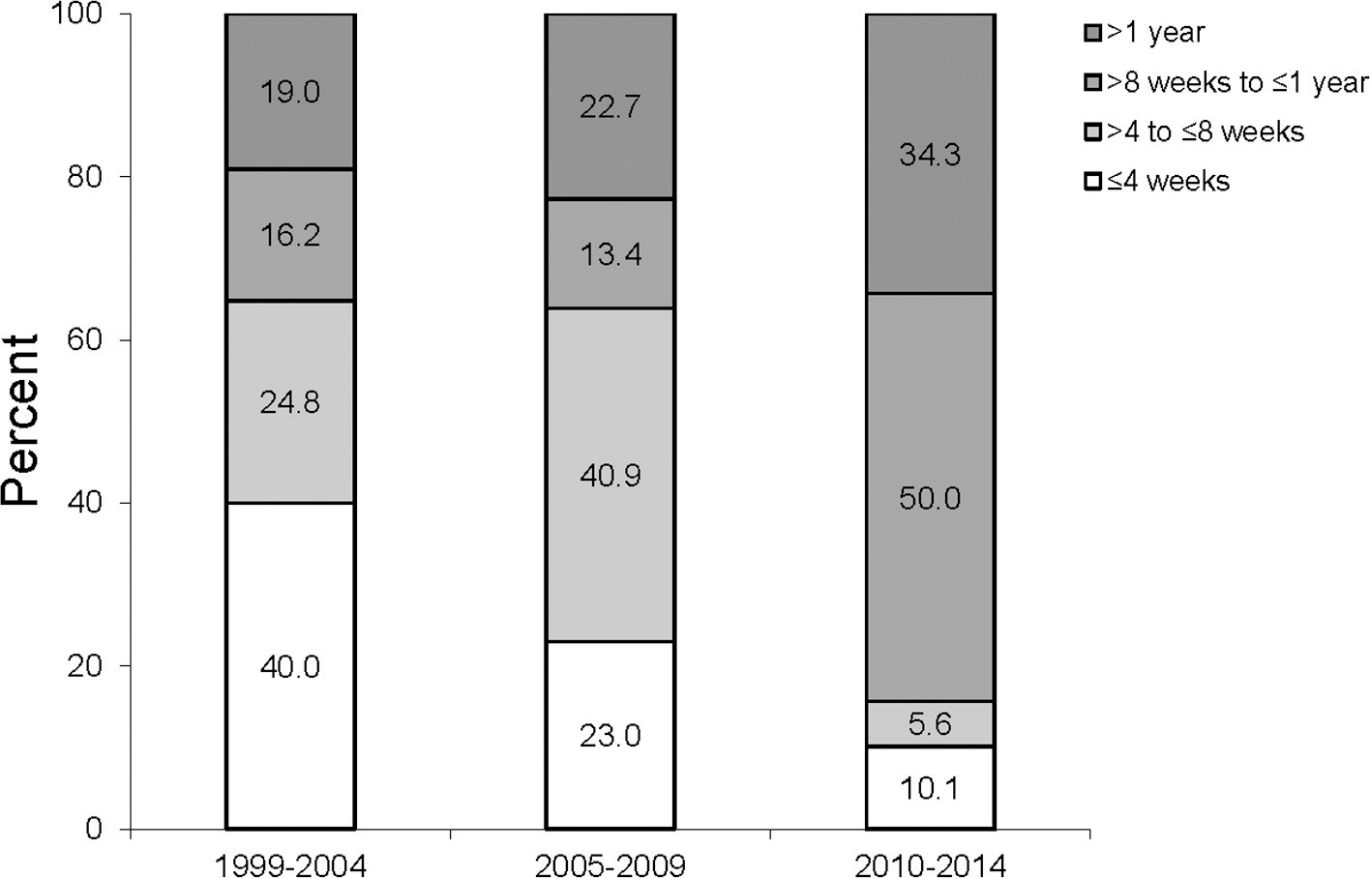
Percentage of interval lengths between the first and the second visit according to the different time periods.

Also the intervals between visits changed (Fig 5). While in 1999–2004 64.8% of the visits occurred within eight weeks, this was in 2010–2014 only in 15.7% the case. Correspondingly, the time between visits was longer than eight weeks in 84.3% of the cases (p≤0.001). Similar results were given for the interval between visit 2 and 3 (data not shown).

### Oral hygiene

**Fig 6:**
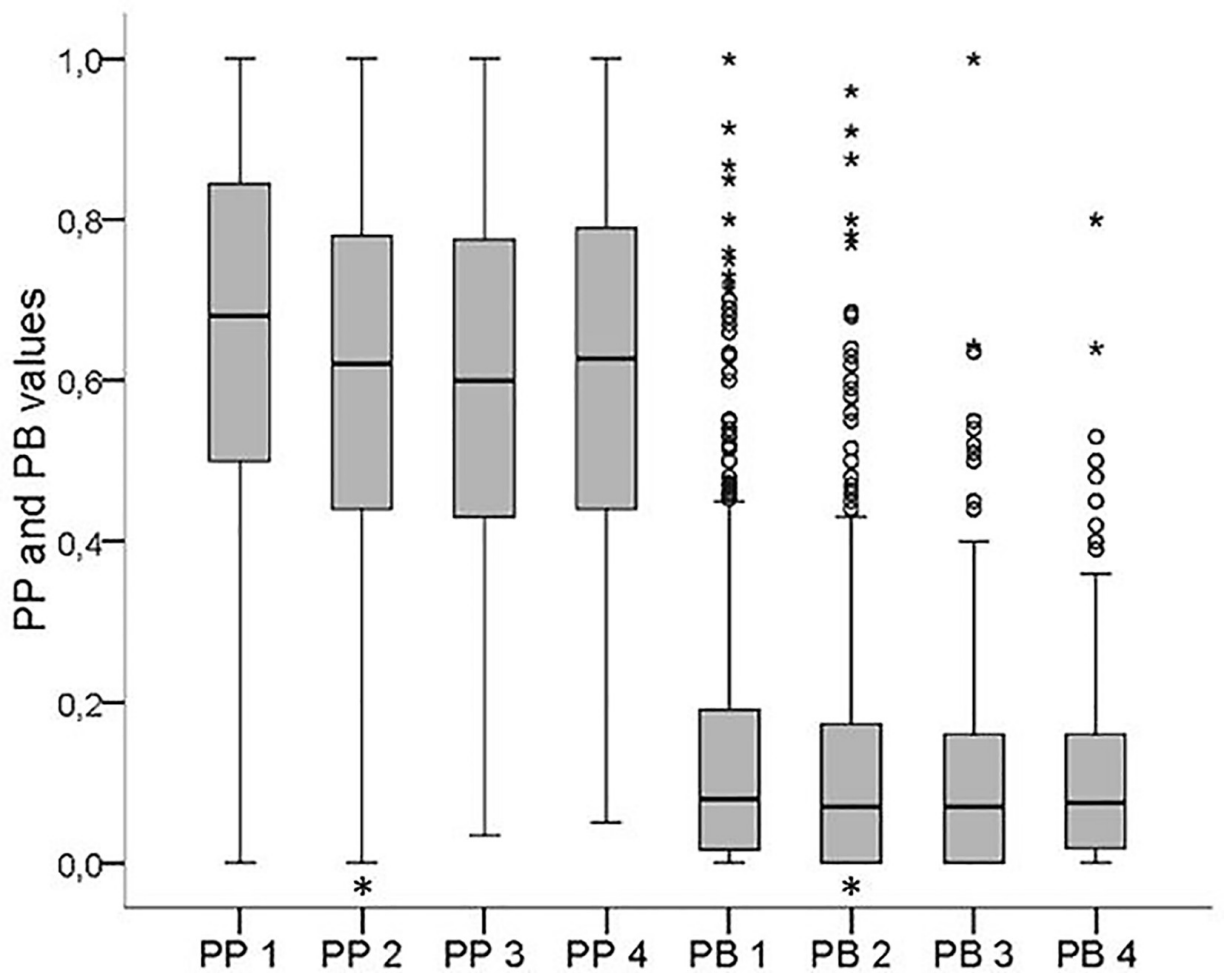
Boxplots of PP and PB values at visits 1–4 (PP 1–4 and PB 1–4 resp.). Asterisks indicate significant differences to the time point before.

The PP values are presented in Fig 6.

At the first visit, patients presented with a wide range of plaque (0.68 (0;1)); There was a small decrease of values at the second visit (0.62 (0;1); p ≤0.001 compared to visit 1), but there was no further improvement over the next visits (visit 2 to 3 and 3 to 4 n.s. each). This was also the case for PB values: at the first visit, the PB was 0.08 (0;1) which decreased significantly at the second visit (0.07 (0;1); p ≤0.001 compared to visit 1) but did not improve further over the next visits (visit 2 to 3 and 3 to 4 n.s. each).

With shorter instruction intervals (≤4 or ≤8 weeks), PP and PB improved significantly (p ≤0.001 each), but this was not the case with longer instruction intervals (≤1 year or longer).

When looking at the different time intervals (Table 2), changes of the PP and PB were more distinct during the first period of the OCP, while there was no significant change of PP and PB values at any time during the last period of the OCP.

**Table 2:** PP and PB values at visits 1–4 (PP 1–4 and PB 1–4 resp.) for the different time periods.

	**PP 1**	**PP 2**	**PP 3**	**PP 4**
**1999–2004**	0.69 (0.07;1.00)	0.57 (0.04;1.00)*	0.58 (0.04;1.00)	0.55 (0.05;1.00)
**2005–2009**	0.61 (0.00;1.00)	0.59 (0.00;1.00)*	0.58 (0.05;1.00)	0.63 (0.01;1.00)
**2010–2014**	0.71 (0.05;1.00)	0.71 (0.00;1.00)	0.72 (0.19;1.00)	0.77 (0.20;1.00)
	**PB 1**	**PB 2**	**PB 3**	**PB 4**
**1999–2004**	0.09 (0.00;1.00)	0.07 (0.00;0.80)*	0.07 (0.00;0.64)	0.07 (0.00;0.80)
**2005–2009**	0.08 (0.00;1.00)	0.07 (0.00;0.88)	0.07 (0.00;1.00)	0.08 (0.00;0.53)
**2010–2014**	0.07 (0.00;1.00)	0.05 (0.00;0.96)	0.08 (0.00;0.51)	0.06 (0.00;0.48)

Data are given as median (min;max)

* indicates significant difference to the preceding visit.

The oral hygiene level at the first visit had a significant impact on the outcome of the next visits (Table 3). Those entering the OCP with poor oral hygiene improved significantly at the next visit, those with a fair oral hygiene improved, but to a lesser extent, and those entering with good oral hygiene worsened significantly. Similar was found for PB values.

**Table 3:** PP and PB values at visits 1–4 (PP 1–4 and PB 1–4 resp.) stratified as to oral hygiene level at the first visit.

	**PP 1**	**PP 2**	**PP 3**	**PP 4**
**good**	0.39 (0.00;0.5)	0.51 (0.00;1.00)*	0.46 (0.04;1.00)	0.49 (0.10;1.00)
**fair**	0.64 (0.51;0.75)	0.59 (0.00;1.00)*	0.55 (0.07;1.00)	0.61 (0.01;1.00)
**poor**	0.89 (0.76;1.00)	0.70 (0.18;1.00)*	0.71 (0.11;1.00)	0.72 (0.11;1.00)
	**PB 1**	**PB 2**	**PB 3**	**PB 4**
**good**	0.00 (0.00;0.03)	0.03 (0.00;0.91)*	0.02 (0.00;0.64)	0.05 (0.00;0.48)
**fair**	0.08 (0.03;0.14)	0.06 (0.00;0.59)	0.09 (0.00;0.50)	0.07 (0.00;0.80)
**poor**	0.25 (0.14;1.00)	0.15 (0.00;0.96)*	0.13 (0.00;1.00)	0.09 (0.00;0.50)

Data are given as median (min;max)

* indicates significant difference to the preceding visit.

**Fig 7:**
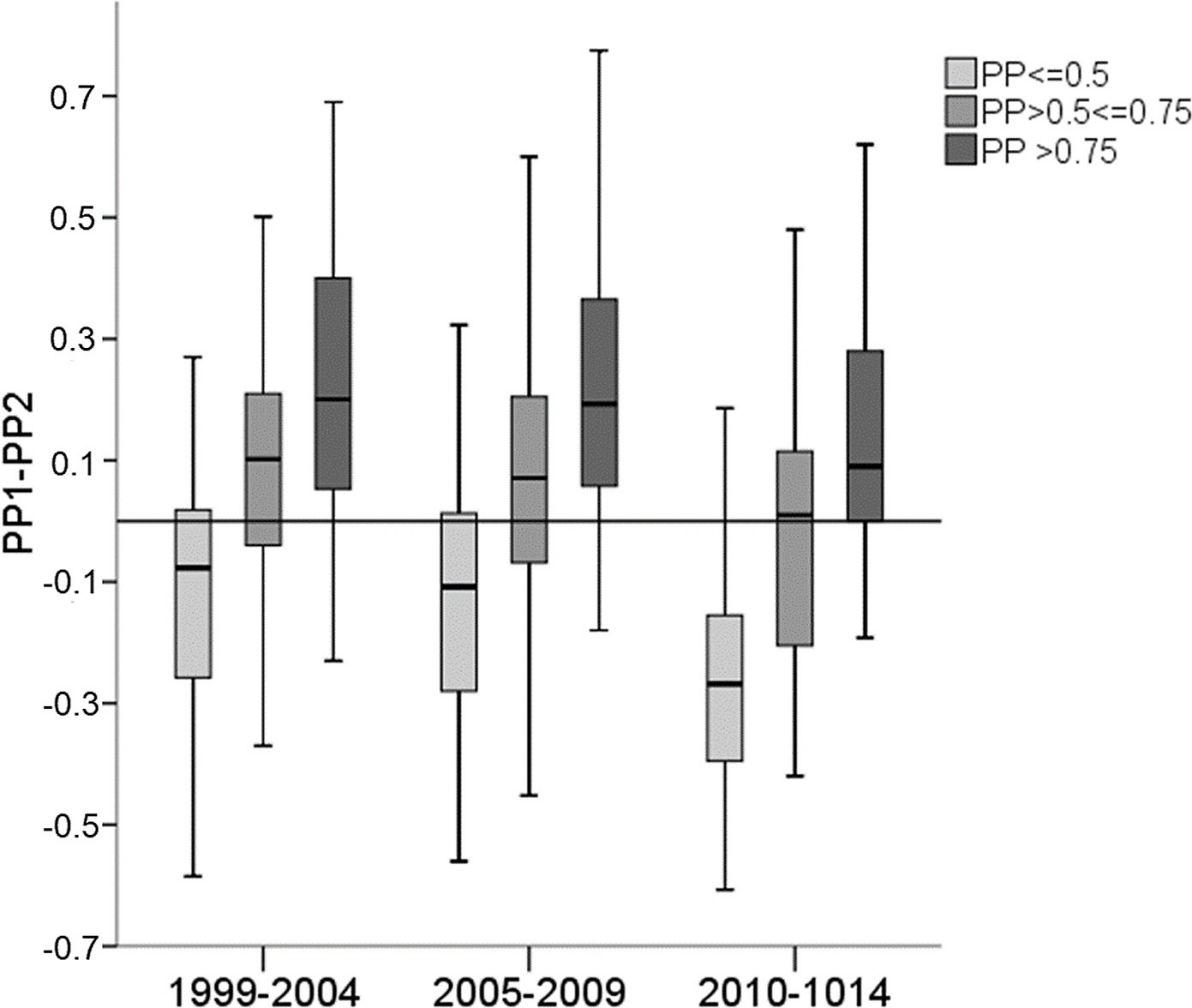
Boxplots of changes of PP values from the first to the second visit (difference of PP 1 –PP 2) according to the time intervals. The line indicates no change, values above the line indicate improved, values below the line indicate worsened PP values.

This picture persisted over time, but the worsening of those with good oral hygiene became more distinct whereas the improvement of those with poor oral hygiene was less distinct (Fig 7).

### Caries

From 747 Patients, D_3/4_MFT and D_3/4_MFS values at the first visit were available. After three years, the number of these patients had decreased to 136.

The median D_3/4_MFT and D_3/4_MFS values of patients entering the OCP were 15 (0;28) and 41 (0;128) resp. Decayed surfaces at the D3/4 level were present in 43.9% of the patients; 36.3% had 1 to 5 lesions, the others had more than 5 lesions (max. 41). Those patients presenting with caries at the first visit had significantly higher PP and PB values than those presenting without caries (PP: 0.74 (0.12; 1.00) versus 0.68 (0.00;1.00); p≤0.001; PB: 0.10 (0.00;1.00) versus 0.07 (0.00;0.9); p≤0.05). Correspondingly, patients with poor oral hygiene at the first visit had significantly more caries lesions than those with fair or good oral hygiene (p≤0.05).

From the 136 patients with a second caries record after 3 years, 32 had developed new lesions by then (D_3/4_T: 1 (1;12)). This was associated with higher PP values compared to those without new lesions (0.82 (0.38;1.00) versus 0.67 (0.13;1.00); p≤0.05) but not with a higher PB value (0.16 (0.00;1.00) versus 0.06 (0.00;0.52); p = 0.07).

## Discussion

In Germany, individual and group-based prevention programmes are covered by both the government and health insurances until age 18. Afterwards, prevention programmes have to be paid by the patient and are performed in private practices. Little is known about the usage of respective programmes by adults, but it seems as if around 20–25% of the patients attend it [20].

A recent consensus report [21] came, inter alia, to the following clinical recommendations: professional toothcleaning should be incorporated in a structured programme including oral hygiene instruction, dietary advice and fluoride application. Furthermore, an individualised, risk-based programme should be designed for each patient. The OCP offered in our clinic was in line with these recommendations as it is a modulate programme covering these items and allowing the tailoring for individual needs.

It was performed by dental students who were assisted by a manual and were supervised by experienced dentists, providing a sufficient degree of quality control. The OCP was free of charge and was not organised on a recall basis; instead, patients were asked to seek for further appointments by themselves as soon as treatments within a semester course were finished. Patients came from routine treatment within our department as well as from outside through word-of-mouth. This means that though the modules were clearly defined and supervised, care was provided by many different students and dentists and addressed the varying clientele of a dental clinic. Thus the OCP may represent the reality in the health care field rather than a strongly controlled setting.

The OCP was attended by patients of a wide range of age and to almost equal parts by women and men. Most of them had appeared to one visit only, whereas approximately 20% used three or more visits. Reasons for the relatively low degree of regular visits may be manifold; e.g. moving to another town, lack of time for treatment in the student’s courses and not being recalled.

Keeping visible plaque and gingival bleeding away is suggested to be a clearly understandable and practical aim in the dental health education of the individual patient. To achieve this goal, the proximal sites of the dentition were our areas of interest. These areas contribute to a greater extent to the plaque covered areas than the oral/vestibular surfaces [18, 22] and are of particular risk for caries. As the Hugoson study [18] has clearly shown, proximal areas are more difficult for patients to clean. After three years of attending prevention programmes, the mean number of oral and vestibular surfaces with plaque was around five per patient while the mean number of proximal surfaces with plaque was 16. The way plaque and bleeding was recorded in the present study was similar to the Hugoson study. It was oriented towards oral hygiene education; therefore, all proximal spaces were observed rather than using established plaque or gingiva indices. The goal was to provide a descriptive measure for demonstrating the patient how plaque and signs of inflammation are related and where oral hygiene deficiencies lie.

Overall, from baseline to the second visit, PP and PB values improved only slightly (by 8.8 and 12.5% resp.). However, it has to be considered that bleeding and plaque were recorded in a dichotomous way and did not quantify the degree of bleeding or amounts of persisting plaque. This means that improvements in oral hygiene beyond complete cleanliness or absence of bleeding could not be recorded. Nevertheless, the outcome of our OCP is much less promising than results from the Axelsson and Hugoson studies. In the latter, the number of proximal surfaces with plaque decreased in the order of around 65% in the intervention groups but also in the control group (approximately by 20%). The reduction of the number of proximal sites with gingivitis was around 40% in the intervention groups but there was almost no improvement in the control group. Amongst other factors like age groups and type of intervention, the main difference between these studies and our OCP is that our patients were not recalled. Another aspect is that patients in the Hugoson study were aware that they participated in a research project, which might have had a distinct impact on the outcome compared to attending a routine dental appointment. This might be indicated by the improvement in the control group, which can be attributed to the Hawthorne-effect.

The initial improvement in PP and PB values found in our study did not improve further with more visits which is similar to results from the Hugoson study. Another finding in our study was that there was a persisting wide span of oral hygiene levels even after several sessions. This indicates that a number of patients was not able to implement sufficient oral hygiene in daily life. The reasons of failing to improve oral hygiene levels may be manifold. A first assumption may be that it is difficult for people to change daily routines persistently what calls for effective psychologically based interventions that might help people to do so. So far, however, there is contradicting evidence that these approaches are effective [23, 24]. The other assumption is that improving plaque levels is a matter of motor capability. Especially the Bass-technique requires dexterity because it comprises of a complex sequence of motions which may be difficult to perform for many patients [25]. Further, adopting a particular brushing technique does not necessarily mean that plaque is removed sufficiently [26]. Video observations from the seventies as well as current publications all reveal that people show an unstructured way of toothcleaning, yet typical motion patterns can be observed. Most subjects tend to move frequently from the left to the right side and spend most of the time brushing the vestibular areas of the dentition [27, 28]. The consistency of findings from the cited video observations indicates the presence of spontaneous and deeply entranced motion patterns. It has been shown that demonstrating systematic toothbrushing and the Bass technique on models or via leaflets can improve toothbrushing behaviour substantially [25]. In view of the above-mentioned observations, however, the question arises, how sustainable such purely rational instruction strategies are. Clearly, further research on the issue is necessary.

An important finding was that the attendance pattern of the OCP changed distinctly over time. In the beginning, patients attended more appointments as well as more different types of modules and chose shorter intervals between the visits. This indicates a more comprehensive interest in issues relevant for oral health. With time, patients increasingly attended only one module, or more often chose the oral hygiene module. Additionally the length of the intervals between visits grew. Reasons for this shift in usage of the OCP might be that patients may feel to be sufficiently informed by other sources like mass media, social media or internet. Also, some of them might lack the time or simply the wish to “consume” a professional toothcleaning.

This change in utilization was associated with a change in the efficacy of the OCP. Overall, there was only a slight improvement of plaque and gingivitis values, but when looking at the different time intervals, it turned out that in the first five years of the OCP where shorter instruction intervals and a more comprehensive use of the different modules occurred, the PP improved significantly by 17% and the PB by 22%. In the last five years of the programme, there was no improvement of these values. This finding would support the assumption that remedial visits in the initial phase of a prevention programme might be necessary to achieve an improvement.

The relevance of the amount of sessions and the time interval between them is unclear. The programme suggested by Axelsson and co-workers [14–17] included recalls to preventive measures once every two to three months over a time period of six years, followed by a need related stratified procedure. Patients at high risk were recalled every three months, whereas those with lower risk were recalled once or twice per year. In a randomised prospective study, those frequent recalls were compared to two different approaches consisting of three visits at two-week intervals and recalls once per year either as an individual- or a group-based preventive programme [18]. After three years, all patients in preventive programmes improved compared to controls, but except for interdental hygiene, which was significantly improved in the group with recalls at two-month intervals compared to the other groups, there was no difference in plaque and gingival values. This might indicate that frequent recalls over years might not be superior over several initial remedial visits followed by recall on a yearly basis.

When looking at the main beneficiaries of the OCP, it was a promising result that those entering with poor oral hygiene at baseline showed the best improvement regarding plaque and gingivitis values (21 and 40% resp.); alarmingly, however, both parameters deteriorated distinctly in those presenting with good oral hygiene at baseline. These effects became more pronounced with time and were most distinct in the last five years of the OCP. It might be speculated that the patients who entered the programme with good oral hygiene tended to transfer responsibility to the professionals assuming that regular professional toothcleaning sessions would take over at least parts of the burden of self-care.

Those with caries data available entered the OCP with a median DMFT of 15 and a median DMFS of 41 which corresponded well with findings from the representative German Oral Health Study [29] showing median DMFT and DMFS values of 14 and 39 resp. for the age group 35–44 years. This indicates that the included group presented with average caries prevalence relative to the caries prevalence observed in Germany. However, almost half of the patients had untreated caries, which is distinctly higher than in the representative study which shows a significant need for preventive measures in this group. As expected, patients with poor oral hygiene had significantly more caries lesions and those who developed new caries within a three years interval, had more proximal plaque than those who stayed caries free for the same time period. This emphasises that the reduction of plaque place an important role in caries prevention, even though there is a widespread use of fluoridated oral care products.

The strength of the present study was that it accumulated data from a large number of patients for more than one decade. It is also an advantage that patients were unselected as the complete archive was analysed and that there was a standardised data sheet for each patient. An exception was caries data, which came from our routine treatment records and was available only for a subgroup of patients.

As with all retrospective studies, data was obtained under routine conditions involving many different examiners without calibration, which represents a weakness of the study. According to this, the performance cannot be controlled and one has to rely on accurate supervision and recordkeeping. In addition, investigators were not blinded; they were aware that there was already a module in advance and knew that previous values might influence performance and recordings during the following module. Thus, this might have caused a bias towards improved index values in follow-up modules compared to the baseline values. Further, there was no control group available. Finally, the OCP was performed in a specific cultural, social, political and legal context. Though the measures provided (oral hygiene, fluoridation and nutrition counselling) are generally accepted pillars of caries prevention, it is obvious that more research is needed to elucidate how these findings can be transposed to different populations. All these aspects do not allow for drawing conclusions as to cause and effect and the results must be interpreted respectively. However, the results presented here provide preliminary data for designing future oral care programmes and prospective studies in the field.

## Conclusions

Several conclusions can be drawn:

- The usage of a preventive programme who’s utilization is left in the patients’ responsibility may change significantly over time, depending on social, economic, educational and cultural conditions in society.
- The order of improvement of oral hygiene was limited in adults attending a non-recall OCP. This means, that not only children and adolescents, but also adults may need a systematic reminder system for maintaining preventive behaviour.
- Changes in the oral hygiene status after attending the OCP were not unidirectional: those with poor oral hygiene were the main beneficiaries of the programme but those with good oral hygiene worsened. This emphasises the need for individualised prevention strategies, perhaps aiming in supporting the patients’ self-responsibility for those who already present good oral hygiene, whereas others might need help to improve their manual skills.
- If the oral hygiene status improved, this occurred within the first two visits; repeated sessions maintained this improvement but contributed little to further advance.
- The interval between initial sessions should not be longer than a few weeks.

Clearly more research is needed when it comes to preventive strategies for adults. Further studies are necessary to accumulate knowledge about oral hygiene skills in this age group and to find ways to improve manual dexterity in order to enable patients to clean their teeth efficiently. Thus, studies on how improved oral hygiene skills can be maintained are needed.

## Supporting information

S1 File Data prevention programme. The file provides the data used in this manuscript.
(XLSX)
